# Deciphering the Mechanism of Bushen Huoxue Decotion on Decidualization by Intervening Autophagy *via* AMPK/mTOR/ULK1: A Novel Discovery for URSA Treatment

**DOI:** 10.3389/fphar.2022.794938

**Published:** 2022-01-24

**Authors:** Xiaoxuan Zhao, Yuepeng Jiang, Jiajie Ren, Yunrui Wang, Yan Zhao, Xiaoling Feng

**Affiliations:** ^1^ Heilongjiang University of Chinese Medicine, Harbin, China; ^2^ School of Basic Medical Sciences, Zhejiang Chinese Medical University, Hangzhou, China; ^3^ Department of Gynecology, The First Affiliated Hospital of Heilongjiang University of Chinese Medicine, Harbin, China

**Keywords:** unexplained recurrent spontaneous abortion, Bushen Huoxue, decidualization, autophagy, AMPK

## Abstract

Impaired decidualization was recognized as one of the crucial pathomechanisms accounting for unexplained recurrent spontaneous abortion (URSA). Currently, the exact molecular mechanism and targeted clinical decision are still in exploration. Bushen Huoxue decoction (BSHXD) has previously been proved effective in treating URSA, but its mechanism remains to be elucidated. This study aimed to explore the regulation mechanism of BSHXD in decidualization from its intervention in autophagy so as to rationalize its potential as a novel therapeutic regime for URSA. Decidua tissues were collected from patients with URSA and healthy pregnant women who underwent legal terminations for non-medical reasons at the first trimester. Besides, cell line T-hESCs was utilized to establish induced decidualization model, and were randomly divided into ESC group, DSC group, 3-MA group, AMPK siRNA group, scrambled siRNA group and AMPK siRNA + BSHXD group. Transmission electron microscopy, Monodansylcadaverine (MDC) assay, qRT-PCR, immunohistochemistry, immunofluorescence and Western blotting were used to evaluate the level of decidualization, autophagy and activation of AMPK signaling pathway in decidua tissues and cell experiments. Experiments on decidua tissues showed that decidualization was impaired in URSA with inhibited autophagy. Besides, pAMPK T172 and pULK1 S556 were decreased, and pmTOR S2448 and pULK1 S757 were increased. Cell experiments showed that the level of autophagy increased during induced decidualization, but when autophagy was inhibited, decidualization was impaired. In addition, AMPK/mTOR/ULK1 affected decidualization by mediating autophagy, and BSHXD improved decidualization through this mechanism. In conclusion, this study clarified that the inhibition of autophagy mediated by AMPK/mTOR/ULK1 was associated with impaired decidualization, and the intervention of BSHXD on this pathological process may be a vital mechanism for its treatment of URSA. This study laid the foundation for further research and application of BSHXD.

## Introduction

Recurrent spontaneous abortion (RSA) is defined as three or more consecutive abortions prior to the 20th week of gestation. Data show that RSA affects approximately 1–5% of reproductive women worldwide (Rai and Regan, 2006; Jaslow et al., 2010). Now several causes and risk factors of RSA have previously been identified, including abnormalities of parental chromosome, uterine or endocrine, infectious causes, thrombotic diseases and immune disorders, etc (Devall and Coomarasamy, 2020; Dimitriadis et al., 2020). However, 50% of RSA cases remain elusive, which is defined as unexplained recurrent spontaneous abortion (URSA). Accumulative evidence suggests that impaired decidualization is a vital pathological mechanism of URSA. Decidualization is defined as the significant morphological, biochemical and functional changes of endometrial stromal cells (ESCs) to decidua stromal cells (DSCs) so as to better adapt to embryo implantation (Ng et al., 2020; Ochoa-Bernal and Fazleabas, 2020). This process is regulated by complex molecular signals, involving huge energy changes and intracellular remodeling, both of which are essential signals for the initiation of autophagy (Popli et al., 2021). Autophagy is a sophisticated and complex cascade of evolutionarily conserved autophagy-related proteins, which is responsible for the degradation of abnormal proteins and organelles in eukaryotic cells, and is significant for energy supply, maintenance of cell homeostasis and promotion of abnormal cell apoptosis (Hurley and Schulman, 2014; Galluzzi and Green, 2019). As decidualization is a highly active biological process involving a large amount of anabolism and catabolism, numerous scholars believe that autophagy is necessary for decidualization. Current studies have confirmed that inhibition of autophagy is associated with impaired decidualization and pregnancy complication (Kim and Lee, 2014; Nakashima et al., 2019), but the regulation mechanism is still perplexing.

AMP activated protein kinase (AMPK) is a key energy sensor and regulates cellular metabolism to maintain energy homeostasis, and one of the mechanisms is to activate autophagy by inactivating mTOR complex-1or by directly phosphorylating ULK1 (Hardie, 2011; Kim et al., 2011). Studies have confirmed that decidualization was accompanied by the activation of AMPK and increased autophagy (Huang et al., 2019; Su et al., 2020), while disordered signal transduction of AMPK signaling pathway has been proved to be related to reduced autophagy and impaired decidualization (Zhang et al., 2021). Therefore, moderate autophagy in response to AMPK activation is critical for pregnancy. However, the correlation between AMPK, autophagy and impaired decidualization in URSA remains unknown.

Various uncertainties in URSA pose great challenges to clinical treatment. Currently, clinicians often use low molecular weight heparin (Pasquier et al., 2015), granulocyte colony-stimulating factor (Eapen et al., 2019), lymphatic immunotherapy (Wang et al., 2016), etc., but there is still a lack of reliable evidence to support the efficacy in improving pregnancy outcome. Therefore, it is urgent to clarify the precise pathological mechanism and explore effective treatments to overcome the intractable medical problem. Traditional Chinese medicine (TCM) has been used for the treatment of RSA for thousands of years, and so far has developed a mature and unique theory for disease diagnosis and treatment. Now, TCM has become the mainstream of reproductive health care in the East Asia by virtue of its prominent efficacy, rich resource, and less toxicity (Yang et al., 2013; Li et al., 2016). Studies have shown that TCM can effectively increase the levels of estrogen and progesterone (Li et al., 2012), improve endometrial receptivity (Yu et al., 2011), and ultimately improve pregnancy outcome (Zhu et al., 2014; Hullender et al., 2015). Shoutai Pill (Chinese Dodder Seed, Himalayan Teasel Root, Chinese Taxillus Twig, and Donkey-hide Glue) is a famous prescription derived from Qing Dynasty, and its efficacy in URSA has been verified by a recent meta-analysis which shows that the addition of Shoutai Pill is superior to western medicine alone in preventing abortion in the first trimester of URSA (Li et al., 2020). Bu-Shen-Huo-Xue decoction (BSHXD) is modified from Shoutai Pill by adding *Astragalus mongholicus Bunge* and *Salvia miltiorrhiza Bunge.* Previous studies *in vitro* or *in vivo* have discovered that BSHXD is capable of preventing miscarriage by playing a comprehensive role in angiogenesis, decuvialization, maternal and fetal immune regulation (Ding et al., 2018; Feng et al., 2020), etc. All these provide objective evidence for the clinical promotion of BSHXD in URSA. However, the complicated molecular regulatory mechanisms of BSHXD on URSA is still in exploration.

**FIGURE 1:**
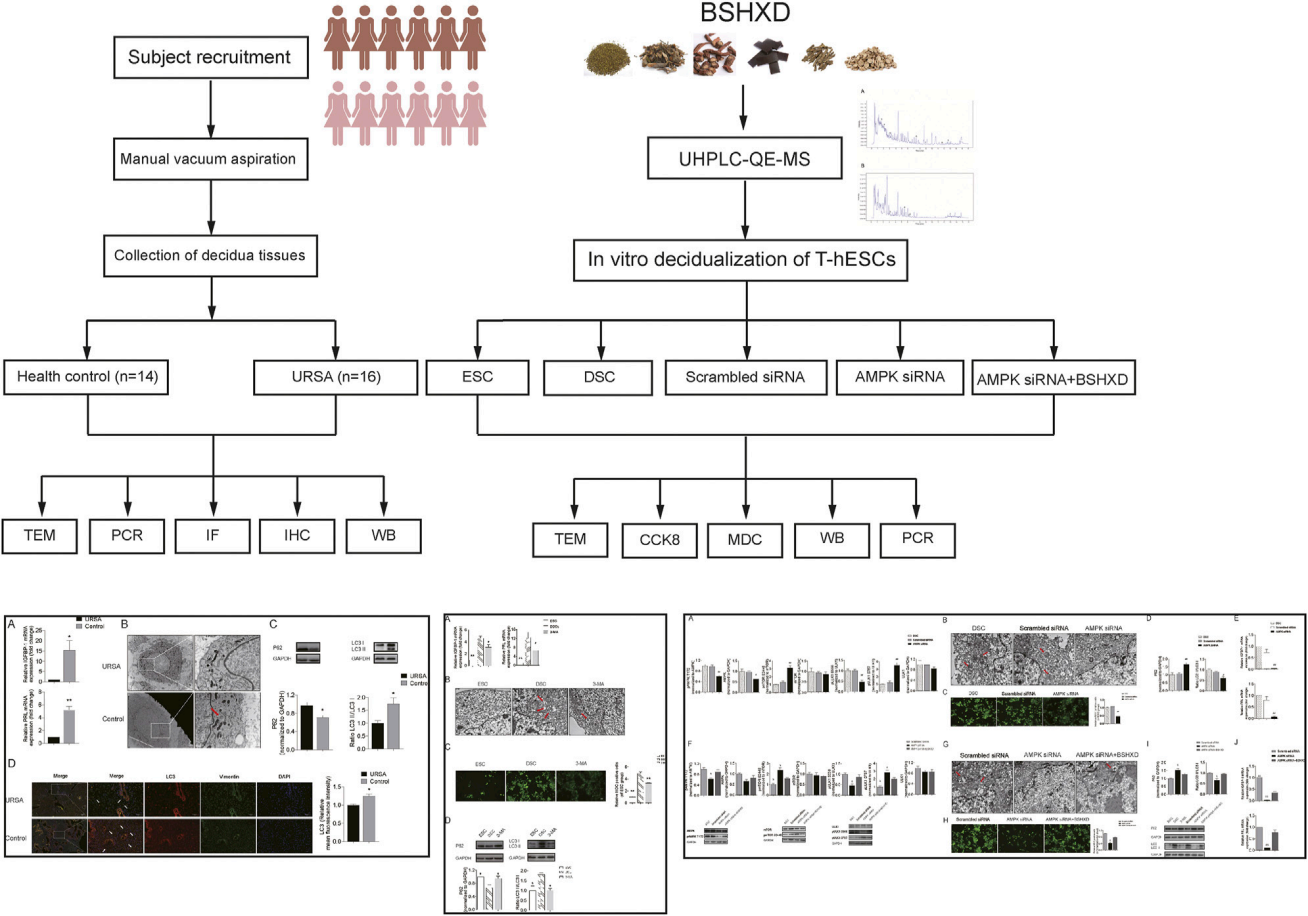
The detailed technical strategy of the current study.

In this study, we first identified the main compounds of BSHXD by UHPLC-QE-MS analysis. And then we determined the level of decidualization, autophagy and the activation of AMPK/mTOR/ULK1 in the decidua tissues of URSA and healthy controls. Besides, the regulation effects of BSHXD on decidualization from the aspects of autophagy *via* AMPK/mTOR/ULK1 were detected in the induced decidualization model. We hypothesized that BSHXD could improve decidualization by promoting autophagy *via* AMPK/mTOR/ULK1 signaling pathway, which may be an important mechanism of BSHXD in the treatment of URSA. The detailed technical strategy of the current study was shown in Figure 1.

## Materials and Methods

### Reagents and Antibodies

DMEM/F12 (D0697; Thermo Fisher), Fetal bovine serum (FBS) (10099141; Gibco), Penicillin (ST488-1; Beyotime), Streptomycin (ST488-2; Beyotime), 8-Br-cAMP (B5386; Sigma), MPA (B1510; APExBIO), 3-MA (3-methyladenine) (S24823; Yuanye Bio-Technology), TritonX-100 (T8200; Solarbio), Vimentin Monoclonal Antibody (60330-1; Proteintech), pULK1 S556 antibody (Bs-3464R; Bioss), p62/SQSTM Polyclonal Antibody (18420-1-AP; Proteintech), LC3 Polyclonal Antibody (14600-1-AP; Proteintech), AMPK*α*2 Rabbit pAb (A7339; ABclonal), pmTOR S2448 Rabbit pAb (AP0094; ABclonal), mTOR Rabbit pAb (A2445; ABclonal), ULK1 Rabbit pAb (A8529; ABclonal), Anti-AMPK alpha 1 (phospho T183) + AMPK alpha 2 (phospho T172) (ab23875; Abcam), pULK1 S757 Rabbit pAb (AP0736; ABclonal), GAPDH (A19056; ABclonal), Goat Anti-Mouse IgG Antibody (Ap124p; Millipore), Goat Anti-Rabbit IgG Antibody (Ap132p; Millipore), Alexa FluorTM 594 Goat Anti-Rabbit IgG (H + L) Antibody (A11037; Thermo), Alexa FluorTM 488 Goat Anti-Mouse IgG (H + L) Antibody (A11029; Thermo), TB Green Premix Ex Taq II (RR820A; TAKARA), PrimeScript RT reagent Kit with gDNA Eraser (RR047A; TAKARA).

### The Preparation of Bushen Huoxue Decoction

BSHXD was composed of six kinds of botanical drugs, and the information and ingredient doses were shown in Table 1. All the six botanical drugs were obtained from the First Affiliated Hospital of Heilongjiang University of Traditional Chinese Medicine (Harbin, China). After weighing and cleaning the above botanical drugs, boil them with pure water (900 ml) and simmer for 30 min. After decocting for twice, mix the liquid and dissolve the donkey-hide glue/gelatin into it. After that, the decoction was filtered and concentrated to paste by rotary evaporator (45°C, −0.1 MPa) and then put into vacuum freeze dryer to prepare lyophilized powder. Finally, the BSHXD lyophilized powder was stored at −20°C for UHPLC-QE-MS analysis and cell experiments.

**TABLE 1:** The composition of Bushen Huoxue Decoction (BSHXD).

TCM materials (Pinyin)	English name	Latin name	Amount (g)
Danshen	The root of red-rooted salvia	Salvia miltiorrhiza Bunge	15
Huangqi	Astragalus membranaceus	Astragalus mongholicus Bunge	15
Sangjisheng	Parasitic loranthus	Taxillus chinensis (DC.) Danser	15
Tusizi	The seed of Chinese dodder	Cuscuta chinensis Lam	15
Xuduan	Teasel root	Dipsacus asper Wall. ex DC.	15
Ejiao	Donkey-hide gelatin	Colla Corii Asini	10

### UHPLC-QE-MS Analysis of Bushen Huoxue Decoction

The UHPLC-QE-MS analysis of BSHXD was performed on a 1290 UHPLC system with a Waters UPLC BEH C18 column (1.7 μm 2.1*100 mm). The column temperature was kept at 55°C and the sample injection volume was 5 μL. The mobile phase consisted of 0.1% formic acid in water (A) and 0.1% formic acid in acetonitrile (B). Linear gradient elution was applied (0–11 min, 85–25% A; 11–12 min, 25–2% A; 12–14 min, 2–2% A; 14–14.1 min, 2–85% A; 14.1–15 min, 85–85% A; 15–16 min, 85–85% A) at a flow rate of 0.5 ml/min. MS detection were performed by using an Q Exactive Focus mass spectrometer (Thermo Fisher Scientific, United States) coupled with an Xcalibur software to maintain the MS and MS/MS data based on the IDA acquisition mode. The operating parameters were as follows: Sheath gas flow rate, 45 Arb; Aux gas flow rate, 15 Arb; Capillary temperature, 400°C; Full ms resolution, 70000; MS/MS resolution, 17500; Collision energy, 15/30/45 in NCE mode; Spray Voltage, 4.0 kV (positive) or −3.6 kV (negative). Identification of chemical compounds from peaks containing MS-MS data was performed by using the secondary mass spectrometry database “BIOTREE PWT database” provided by Shanghai BIOTREE biotech Co., Ltd. and the corresponding cleavage law matching method.

### Subjects and Sample Collection

The URSA group consisted of 16 women who had experienced twice or more miscarriage at early pregnancy, and the certain cause of miscarriage had been ruled out, such as endocrine, anatomical or genetic abnormalities, infection and other factors. The control group included 14 healthy women who voluntarily terminated pregnancy at an early stage due to non-medical factors. Besides, they had at least one healthy child without abortion history. The fetal heart rate of this pregnancy was normal identified by ultrasound examination, and the fetal development was consistent with the gestational age. Besides, all the subjects should meet the following requirements: 25–40 years old, 6–9 weeks of gestation, BMI between 18-29, normal menstrual history, no serious comorbidities and no bad habits such as smoking or drinking. There were no statistical differences in age, gestation time, and BMI between the two groups, as shown in Supplementary Table S1.

In our practice, curettage was routinely performed in both URSA and the control group, and the decidua tissue samples were obtained immediately after the operation. The tissue was washed with cold PBS and divided into three parts. All tissues used for quantitative real-time polymerase chain reaction (qRT-PCR) and Western blotting analysis were cryopreserved at −80°C. Transmission electron microscopy (TEM) samples were immersed in 2.5% glutaraldehyde. Immunohistochemical and immunofluorescence samples were fixed with 4% paraformaldehyde.

### Cell Culture and *in vitro* Decidualization of T-hESC

As described in the previous literature (Zhang et al., 2016; Huang et al., 2019), T-hESC (human telomerase reverse transcriptase-immortalized endometrial stromal cells, ATCC, CRL- 4003) were cultured in phenol red-free DMEM/F12 medium containing 10% charcoal-stripped fetal bovine serum (CS-FBS), 100U/mL penicillin, 100 μg/ml streptomycin with 0.5 mM 8-Br-cAMP and 1 uM MPA to induce decidualization *in vitro*. The induction process lasted for 6 days with medium changing every 2 days. The decidualization was evaluated by morphological phenotype and the increased level of decidual markers PRL mRNA and IGFBP1 mRNA (Huang et al., 2020).

### CCK-8 Assay for the Selection of Optimal Concentration and Time of Bushen Huoxue Decoction Treatment

The final concentration of BSHXD was set at 0.25 mg/ml, 0.5 mg/ml, 1 mg/ml, 2 mg/ml and 4 mg/ml, and the cell viability was respectively determined at 0, 24, 48 and 72 h after treatment by the CCK-8 assay kits in accordance with the manufacturer’s instructions. The absorbance at 450 nm was measured using a microplate reader (PerkinElmer, Waltham, MA, United States).

### siRNA Transfection

Briefly, T-hESC was inoculated in 24-well plates with 2×10^5^ cells/well. After about 40% of the adherent cells were fused, three small interfering RNA (siRNA) oligos specific for human AMPK (stB0004655C genOFFTM st-h-PRKAA1_003, siG08121512363703 si-h-PRKAA1_002, siG000005562C si-h-PRKAA1_103, RiboBio, China) or scrambled siRNA (siN0000001-1-5 siR NC #1, 5nmol, RiboBio, China), were transfected into cells by using transfection reagents riboFECT™ CP Reagent (RiboBio, China) according to the manufacturer’s instructions. The final concentration of each siRNA was 50 nM. And the T-hESCs were transfected on day 0 and day 4 after MPA and 8-Br-cAMP treatment (Shukla et al., 2019)*.* The best specific knockdown efficacy was selected from the three interference sequences by Western blot and was used for subsequent experiments.

### Cell Processing and Grouping

T-hESCs were randomly divided into ESC group (control group), DSC group (treatment of 1uM MPA +0.5 mM 8-Br-camp for 6 days), 3-MA group (treatment of 10 mM 3-MA for 24 h on the fourth day of decidualization), AMPK siRNA group (transfected with 50 nM AMPK siRNA on day 0 and day 4 of decidualization), scrambled siRNA group (transfected with 50 nM scrambled siRNA on day 0 and day 4 of decidualization) and AMPK siRNA + BSHXD group (BSHXD was given on the fourth day of decidualization, and the concentration and time were determined based on CCK-8 assay).

### Transmission Electron Microscopy

The decidua tissue or the cells digested by trypsinase were rapidly fixed in precooled 2.5% glutaraldehyde solution for 24 h, and then washed with PBS and transferred to 1% osmium and 1.5%K_3_ [Fe(CN)_3_] for 1 h. The samples were immersed in a dioxy solution of 2% acetic acid at 4°C overnight, and then experienced dehydration in gradient ethanol solution and soaked in different proportions of pure acetone and embedding agent. The samples were polymerized at 60°C, and made into ultra-thin sections. Uranyl acetate and lead citrate (3%) were utilized to stain the sections. The formation of autophagosomes was observed under a electron microscope (Hitachi, Japan).

### Monodansylcadaverine (MDC) Staining

After the cells in each group were treated for corresponding time, discard the supernatant cleaned with 1×Wash Buffer. Each cell was added with 90 μL 1×Wash Buffer and 10 μL MDC staining solution, and incubated in darkness at room temperature for 30 min. After incubation, the cells were washed with 1×Wash Buffer for 2 times and observed under a fluorescence microscope immediately.

### qRT-PCR

Total RNA from decidual tissue or cells were isolated by RNAiso Plus (Takara Bio, Japan) according to the manufacturer’s recommendations. 1 μg RNA was reverse transcribed using PrimeScript™ RT reagent Kit with gDNA Eraser (Takara Bio, Japan). Relative mRNA levels of PRL, IGFBP1 and *β*-actin were determined by qRT-PCR using TB Green Premix Ex Taq II(Takara Bio, Japan). The primer sequences used for real-time PCR were as follows:

PRL-Human-F:ACCTCTCCTCAGAAATGTTCAGCGA; PRL-Human-R:TCTGTTGGGCTTGCTCCTTGTCTT; IGFBP1-Human-F:AAAGCCCAGAGAGCACGGAGATAA;

IGFBP1-Human-R: ATG​GCG​TCC​CAA​AGG​ATG​GAA​TGA.

### Immunohistochemistry

The decidual tissues were fixed with 4% paraformaldehyde, and then dehydrated, and embedded in paraffin. Paraffin sections (5 μm) were prepared, dewaxed, and rehydrated in ethanol. After antigen repair, the slides were blocked with 3% hydrogen peroxide for 10min to inhibit endogenous peroxidase activity, and then nonspecific binding sites were blocked with 5% bovine serum albumin for 30 min. After that, sections were incubated overnight with rabbit anti-pAMPK T172 antibody and anti-pmTOR S2448 antibody at 4°C overnight, and anti-pULK1 S556, anti-pULK1 S757 then incubated with goat anti-rabbit immunoglobulins for 1 h at room temperature. Afterwards, sections were subsequently re-stained with hematoxylin. Finally, the tissue sections are dehydrated and sealed, and images were captured with an optical microscope.

### Immunofluorescence

The decidual tissues were fixed with 4% paraformaldehyde in PBS for 10 min, and then infiltrated with 200 μL 0.3% Triton X-100 for 10 min. After that, the cells were then blocked with 5% bovine serum albumin (BSA) for 30 min, stained with primary antibody overnight (pAMPK*α*2 T172, 1:250; LC3, 1:200; Vimentin, 1:200), and incubated with secondary antibody for 2 h (Alexa FluorTM 488 Goat Anti-Mouse IgG (H + L) Antibody, 1:250; Alexa FluorTM 594 Goat Anti-Rabbit IgG (H + L) Antibody, 1:250). At last, DAPI was utilized to counterstain the decidual tissues and then the tissues were observed under the fluorescence microscope.

### Detection of Protein Expression by Western Blotting

The expressions of p62, LC3 in decidual tissues and p62, LC3, AMPK*α*2, pAMPK*α*2 (T172), ULK1, pULK1 (S757), pULK1 (S556), pmTOR (S2448), mTOR in cells were detected by Western blotting. First, lysates of decidua tissue or cells were prepared in high efficiency cell tissue rapid lysis buffer (RIPA; Beyotime, China). Then, protein concentration was determined by the BCA Protein Detection Kit (Beyotime, China). After that, sample proteins were isolated by sodium dodecyl sulphate-polyacrylamide gel elec-trophoresis and then transferred to polyvinylidene difluoride (PVDF) membrane at 100V, 65 min. And then the PVDF blots were incubated with primary antibodies at 4°C overnight: p62 (1:1000), LC3 (1:1000), AMPK*α*2 (1:1000), pAMPK*α*2 T172 (1:1000), ULK1 (1:1000), pULK1 S757 (1:1000), pULK1 S556 (1:1000), pmTOR S2448 (1:1000), mTOR (1:1000). Subsequently, the membranes were washed and further incubated with secondary antibodies. Finally, the PVDF membrane was immersed in ECL luminescent solution and scanned by the developer after 2 min of shading.

### Statistical Analysis

All results were presented as the mean ± SEM and analyzed by GraphPad Prism 8 (GraphPad Software, San Diego, CA, United States). Statistical analyses were performed by two-tailed unpaired *t* test or one-way ANOVA with Dunnett’s multiple comparisons. A value of *p* < 0.05 was regarded as statistically significant.

## Results

### Components Analysis of Bushen Huoxue Decoction by UHPLC-QE-MS

**FIGURE 2:**
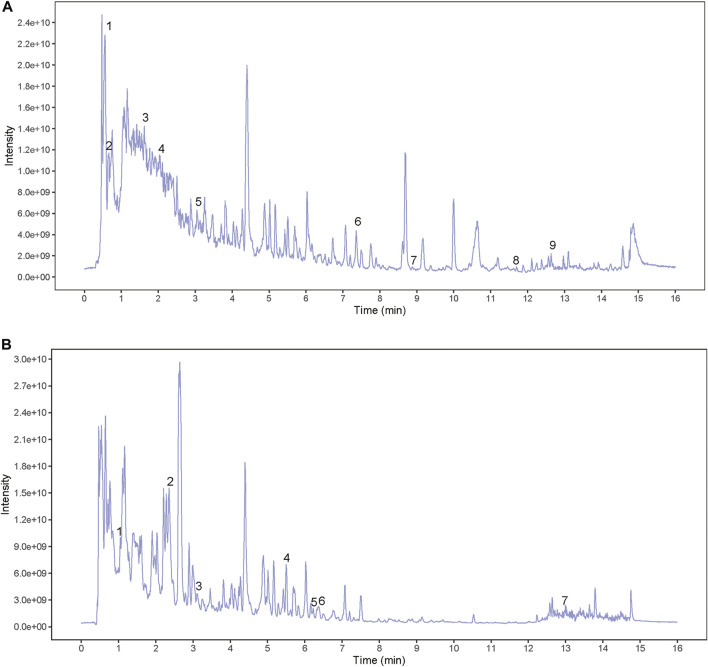
The total positive and negative ion chromatograms of BSHXD. **(A)** The total positive ion chromatograms of BSHXD; **(B)** The total negative ion chromatograms of BSHXD.

In order to identify the main components of BSHXD, UHPLC-QE-MS analysis was applied. The total positive (Figure 2A) and negative (Figure 2B) ion chromatograms of BSHXD demonstrated the chemical composition of all compounds. Major chemical compositions were further identified by using the BIOTREE PWT database of BIOTREE Biotechnology Co., Ltd. (Shanghai, China). Sixteen compounds were distinguished, and the information obtained by secondary mass spectrometry were shown in Table 2 and Supplementary Figure S1.

**TABLE 2:** The identification of chemical constituents of BSHXD by UHPLC-QE-MS. **(A)** The positive ion mode. **(B)** The negative ion mode.

NameEN	Composite score	InChIKey	Simplified molecular input line entry system (SMILES)	Formula	name	Class	Mzmed	Rtmed	z	ppm	ms2Adduct
1. Proline	0.981567538461539	ONIBWKKTOPOVIA-BYPYZUCNSA-N	C1C[C@H](NC1)C(=O)O	C5H9NO2	M116.070T32.766	Alkaloids	116.0703082	32.7661	467625163.8	2.655620922	[M + H]+
2. Valine	0.972773461538462	KZSNJWFQEVHDMF-BYPYZUCNSA-N	O=C(O)C(N)C(C)C	C5H11NO2	M118.086T36.716	Alkaloids	118.0861079	36.71615	143002818.8	0.914104799	[M + H]+
3. Hyperoside	0.976750076923077	OVSQVDMCBVZWGM-DTGCRPNFSA-N	OC[C@@H](O1)[C@H](O)[C@H](O)[C@@H](O)[C@@H]1OC(C(=O)3)=C(Oc(c4)c(c(O)cc(O)4)3)c(c2)cc(O)c(O)c2	C21H20O12	M465.102T94.667	Flavonoids	465.1016295	94.6669	363599440.1	0.796494513	[M + H]+
4. Quercitrin	0.984994846153846	OXGUCUVFOIWWQJ-HQBVPOQASA-N	Oc(c4)c(O)cc(c4)C(O1)=C(O[C@H](O3)[C@H](O)[C@H](O)[C@@H](O)[C@H](C)3)C(=O)c(c(O)2)c (cc(O)c2)1	C21H20O11	M449.108T121.216	Flavonoids	449.1081521	121.216	265759390.9	2.565339446	[M + H]+
5. Salvianolic acid C	0.728436230769231	GCJWPRRNLSHTRY-VURDRKPISA-N	C1=CC(=C(C=C1CC(C(=O)O)OC(=O)C=CC2=C3C=C(OC3=C(C=C2)O)C4=CC(=C(C=C4)O)O)O)O	C26H20O10	M493.113T180.205	Phenylpropanoids	493.1129581	180.205	12665219.21	0.08506921	[M + H]
6. Dihydrotanshinone I	0.611710153846154	HARGZZNYNSYSGJ-UHFFFAOYSA-N	CC1COC2=C1C(=O)C(=O)C3=C2C=CC4=C(C=CC=C43)C	C18H14O3	M279.101T442.074	Quinones	279.1014183	442.074	1757127057	1.498589121	[M + H]
7. Tanshinone IIA	0.963680153846154	HYXITZLLTYIPOF-UHFFFAOYSA-N	CC1=COC2=C1C(=O)C(=O)C3=C2C=CC4=C3CCCC4(C)C	C19H18O3	M295.133T536.366	Diterpenoids	295.1329611	536.366	5479658.754	0.131732182	[M + H]
8. Ursolic acid	0.843185538461538	WCGUUGGRBIKTOS-GPOJBZKASA-N	CC1CCC2(CCC3(C(=CCC4C3(CCC5C4(CCC(C5(C)C)O)C)C)C2C1C)C)C(=O)O	C30H48O3	M457.367T698.234	Terpenoids	457.3673024	698.234	3533652.684	0.661213336	[M + H]
9. Cryptotanshinone	0.974581230769231	GVKKJJOMQCNPGB-JTQLQIEISA-N	CC1COC2=C1C(=O)C(=O)C3=C2C=CC4=C3CCCC4(C)C	C19H20O3	M297.149T755.898	Diterpenoids	297.1485799	755.898	16991913.25	1.951405008	[M + H]
1. Chlorogenic acid	0.678918615384615	CWVRJTMFETXNAD-JUHZACGLSA-N	O=C(O)C1(O)CC(O)C(O)C(OC(=O)C=CC2=CC=C(O)C(O)=C2)C1	C16H18O9	M353.087T61.398	Phenylpropanoids	353.0871462	61.3977	830572123.4	0.414167858	[M-H]-
2. Salvianolic acid A	0.929191769230769	YMGFTDKNIWPMGF-UCPJVGPRSA-N	C1=CC(=C(C=C1CC(C(=O)O)OC(=O)C=CC2=C(C(=C(C=C2)O)O)C=CC3=CC(=C(C=C3)O)O)O)O	C26H22O10	M493.114T141.447	Phenylpropanoids	493.1141289	141.447	2733433945	0.261335365	[M-H]
3. Quercetin	0.997640307692308	REFJWTPEDVJJIY-UHFFFAOYSA-N	C1=CC(=C(C=C1C2=C(C(=O)C3=C(C=C(C=C3O2)O)O)O)O)O	C15H10O7	M301.035T188.792	Flavonoids	301.0352775	188.792	344246998.2	0.921724202	[M-H]
4. Astragaloside IV	0.954460923076923	QMNWISYXSJWHRY-CSXKERSZSA-N	CC1(C(CCC23C1C(CC4C2(C3)CCC5(C4(CC(C5C6(CCC(O6)C(C)(C)O)C)O)C)C)OC7C(C(C(C(O7)CO)O)O)O)OC8C(C(C(CO8)O)O)O)C	C41H68O14	M829.459T325.802	Terpenoids	829.4593902	325.802	159589303.1	3.146435485	[M + HCOO]
5. Soybean saponin fraction B1	0.953320923076923	PTDAHAWQAGSZDD-UHFFFAOYNA-N	CC1OC(OC2C(O)C(O)C(CO)OC2OC2C(O)C(O)C(OC2OC2CCC3(C)C(CCC4(C)C3CC=C3C5CC(C)(C)CC(O)C5(C)CCC43C)C2(C)CO)C(O)=O)C(O)C(O)C1O	C48H78O18	M941.511T371.947	Terpenoids	941.5113036	371.947	121400159.7	0.322480417	[M-H]-
6. Astragaloside II	0.951037	AYWNHWGQTMCQIV-PENCHUSISA-N	CC(=O)OC1C(C(COC1OC2CCC34CC35CCC6(C(C(CC6(C5CC(C4C2(C)C)OC7C(C(C(C(O7)CO)O)O)O)C)O)C8(CCC(O8)C(C)(C)O)C)C)O)O	C43H70O15	M871.471T383.757	Terpenoids	871.4710616	383.757	86162854.18	1.218139411	[M + HCOO]
7. Loganic acid	0.970480076923077	JNNGEAWILNVFFD-CDJYTOATSA-N	CC1C(CC2C1C(OC=C2C(=O)O)OC3C(C(C(C(O3)CO)O)O)O)O	C16H24O10	M375.130T779.793	Iridoids	375.1295698	779.793	14527943.37	1.5188469	[M-H]

### Autophagy was Inhibited in the Decidua Tissues of Unexplained Recurrent Spontaneous Abortion With Impaired Decidualization

**FIGURE 3:**
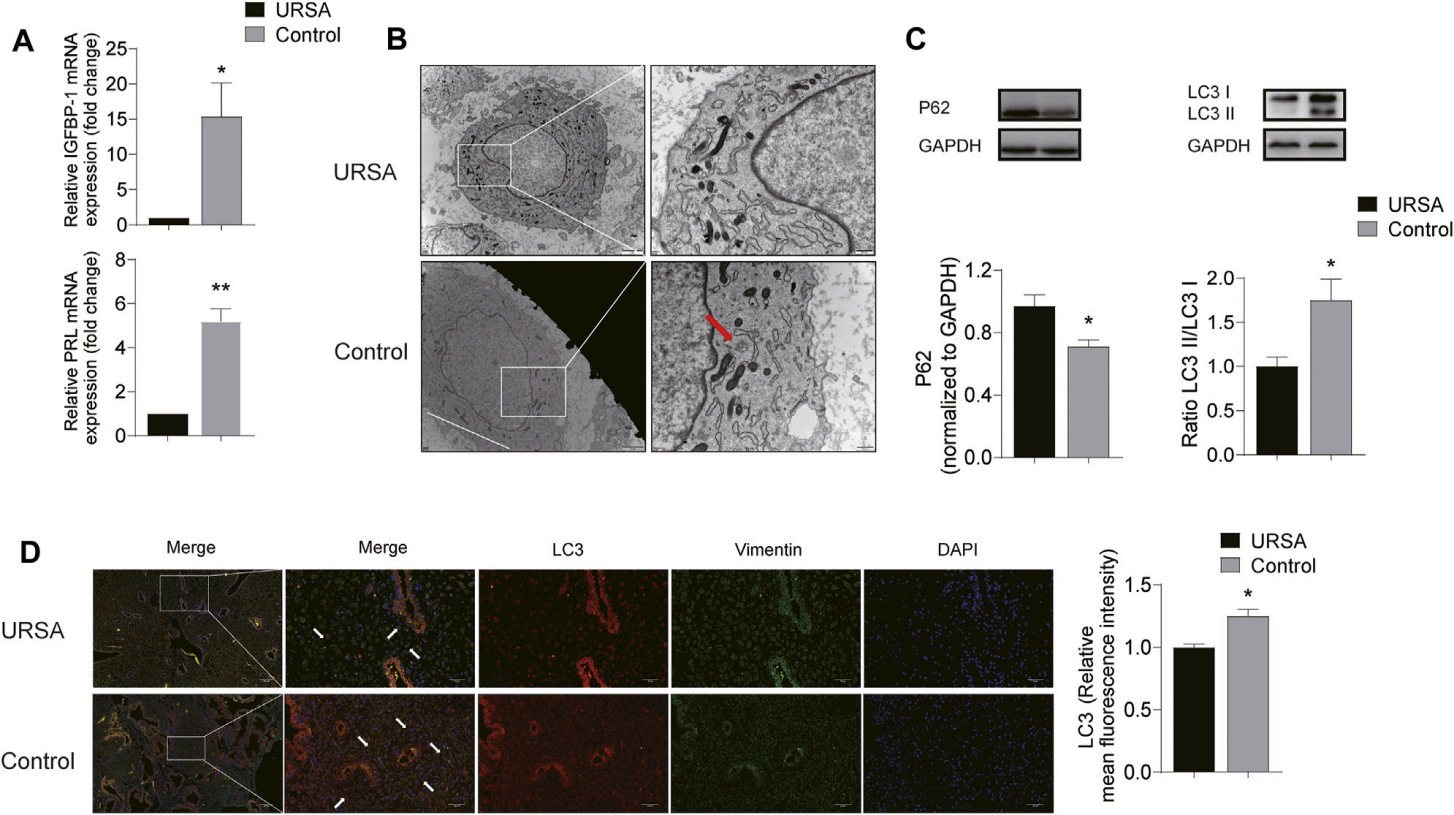
Autophagy was inhibited in the decidua tissues of URSA with impaired decidualization. **(A)** mRNA of IGFBP1 and PRL in the URSA group and control group; **(B)** The autophagosomes in the URSA group and control group observed by TEM (scale bar: 2 μm and 500 nm); **(C)** The expression levels of LC3II/LC3I and p62 in the decidua tissue used by Western blot; **(D)** Immunofluorescence co-location of LC3 and vimentin, and the expression of LC3 (scale bar: 200 μm and 50 um). *p < 0.05 vs. URSA group.

Decidualization is a secreted phenotype transition from ESCs to DSCs, characterized by increased level of PRL and IGFBP1, which have been widely used as specific markers of decidualization (Tang et al., 1993; Krikun et al., 2004). Thus, we assessed the decidualization condition of URSA patients by detecting the mRNA level of PRL and IGFBP1 in decidua tissues. Results showed that the mRNA levels of IGFBP1 and PRL in the URSA group were significantly lower than the control group (*p* < 0.05 and *p* < 0.01, respectively) (Figure 3A), indicating impaired decidualization in URSA.

Considering that autophagy inhibition was closely related to impaired decidualization (Rhee et al., 2016), and that the molecular biological pathway predicted by network pharmacology could also directly affect autophagy, we compared the autophagy level in the two groups. Autophagosomes defined by TEM is regard as the direct evidence and gold standard for autophagy. We observed autophagy at different stages from different sections under different fields in the control group, while the number of autophagosomes in URSA group was reduced or even absent in multiple visual fields (Figure 3B). To further quantify the level of autophagy in the two groups, western blot was used to detect the expression levels of LC3II/LC3I and p62, the two of which can be used to evaluate the status of autophagy flux. The results showed that the LC3II/LC3I ratio in the URSA group decreased significantly, while p62 increased (Figure 3C), indicating the decreased level of autophagy in URSA (*p* < 0.05). Considering that DSCs are the main components of decidua tissue, immunofluorescence co-location analysis of LC3 and vimentin (a marker of DSCs) was performed. And the results showed that LC3 and vimentin were colocalization, indicating that LC3 was expressed in DSCs. In addition, the intensity of LC3 fluorescence in URSA group was significantly lower than that in the control group (*p* < 0.05) (Figure 3D), suggesting that the decreased autophagy level of DSCs contributed to the autophagy inhibition in the decidua of URSA.

### AMPK/mTOR/ULK1 May Be Associated With the Decreased Autophagy in Decidual Tissue of Unexplained Recurrent Spontaneous Abortion

**FIGURE 4:**
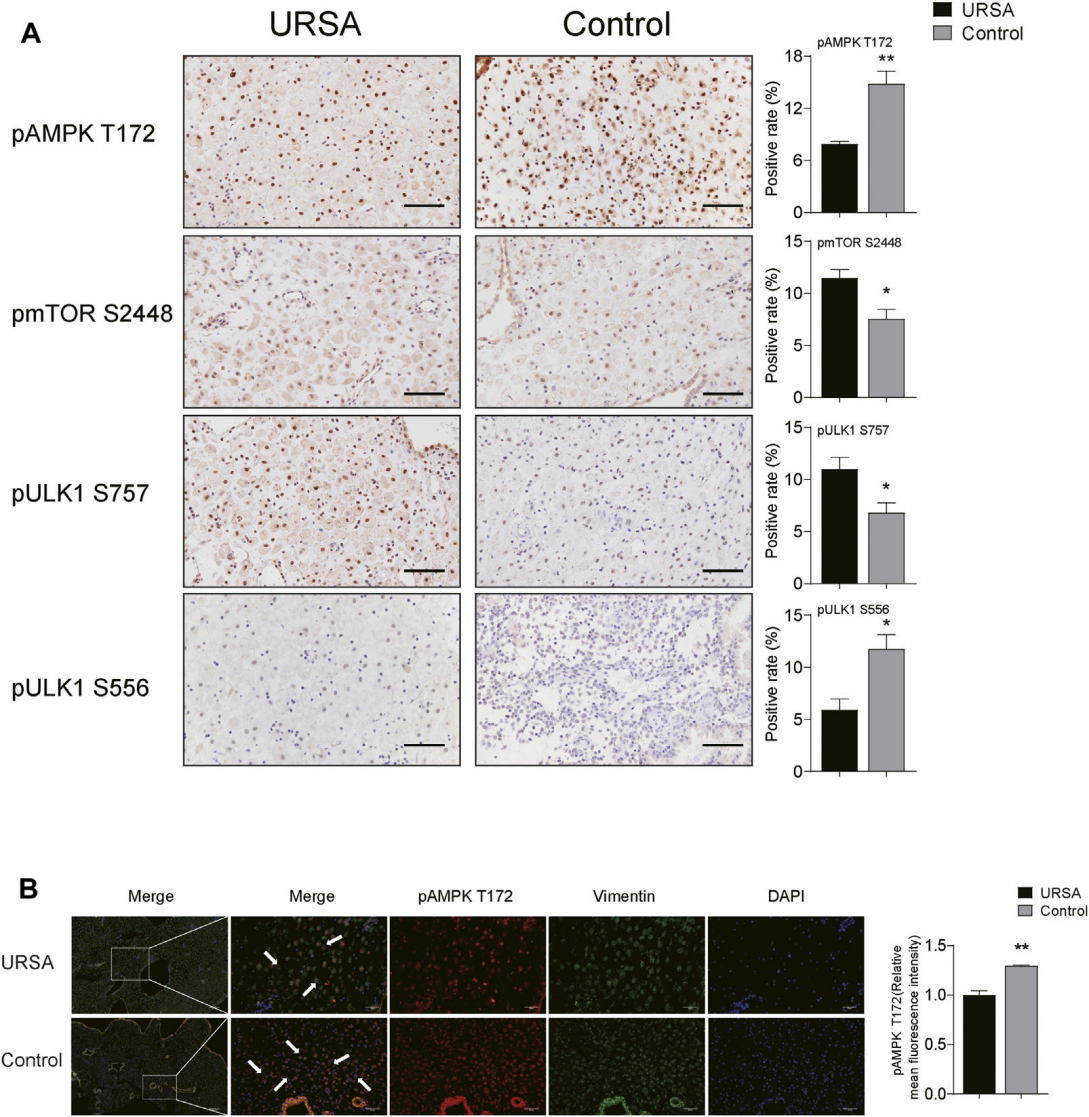
AMPK/mTOR/ULK1 may be associated with the inhibition of autophagy. **(A)** The expression of pAMPK, pmTOR and pULK1 in decidua tissues of the two groups by immunohistochemistry (scale bar: 50 μm); **(B)** Immunofluorescence co-location of pAMPK and vimentin, and the expression of pAMPK (scale bar: 200 and 50 μm). *p < 0.05 vs. URSA group; **p < 0.01 vs. URSA group.

Considering that the co-targets predicted by network pharmacology are mainly enriched in AMPK signaling pathway, and AMPK can promote autophagy by directly promoting phosphorylation of ULK1 S556 or by inhibiting phosphorylation of pmTOR S2448 and pULK1 S757. Therefore, we detected the expression of pAMPK, pmTOR and pULK1 in decidua tissues of the two groups by immunohistochemistry. The results displayed that the positive area of pAMPK T172 and pULK1 S556 in URSA group was lower than that in control group (*p* < 0.01 and *p* < 0.05, respectively), while the positive area of pmTOR S2448 and pULK1 S757 in URSA was significantly increased (*p* < 0.05) (Figure 4A). Considering that the decreased level of autophagy in DSCs in URSA, immunofluorescence co-location analysis of pAMPK and vimentin was conducted to determine the activation degree of AMPK in DSCs between the two groups. The results showed that the fluorescence distribution of pAMPK and vimentin was colocalization, and the fluorescence intensity in the URSA group was significantly lower than that in the control group (*p* < 0.01) (Figure 4B), suggesting that the activation of AMPK in DSCs of URSA was decreased. Taken together, the above experiments suggested that the reduced autophagy of DSCs in URSA may be related to the down-regulation of AMPK/mTOR/ULK1, but the causal relationship between the two needs to be further confirmed *in vitro* experiments.

### CCK-8 Assay

In order to screen out the optimal concentration and intervention time of BSHXD on T-HESC, CCK-8 assay was utilized to calculate the cell viability of each group treated by different concentrations of BSHXD under different time. The results showed that the cell activity was highest when 0.25 mg/ml BSHXD was administered for 48 h, and this intervention was used in the follow-up experiment, as shown in Supplementary Figure S2.

### Selection of the AMPK siRNA With Best Knockdown Efficacy

In order to select AMPK siRNA oligos with the best knockdown efficacy, three siRNA were respectively transfected into T-hESCs, and the results were shown in the Supplementary Figure S3. The AMPK and pAMPK/AMPK levels were significantly decreased in all the three siRNA sequences when compared with the scrambled siRNA, and the knockdown efficacy of AMPK siRNA2 was the most significant, which was used for subsequent experiments.

### Autophagy was Increased in Decidualization *in vitro*, While Autophagy Inhibition Led to Impaired Decidualization

**FIGURE 5:**
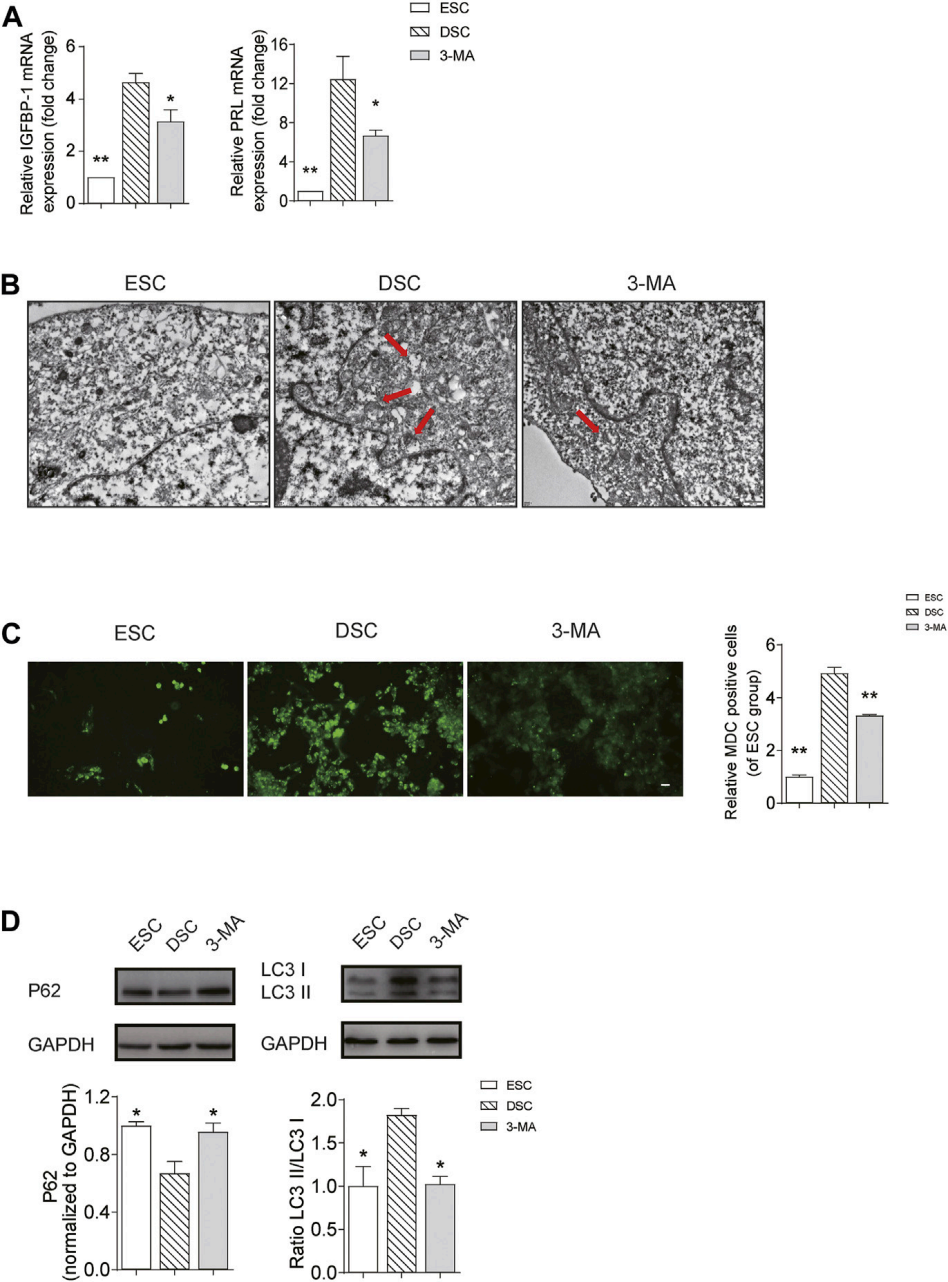
Autophagy was increased in decidualization *in vitro*, while inhibition of autophagy can impair decidualization. **(A)** The mRNA levels of IGFBP1 and PRL in the ESC, DSC and 3-MA group; **(B)** The autophagosomes in the ESC, DSC and 3-MA group observed by TEM (scale bar: 500 nm); **(C)** The autophagy level in the ESC, DSC and 3-MA group observed by MDC assay (scale bar: 50 μm); **(D)** The expression levels of LC3II/LC3I and p62 in the ESC, DSC and 3-MA group used by Western blot. *p < 0.05 vs. DSC group; **p < 0.01 vs. DSC group.

To further reveal whether autophagy inhibition is the cause of impaired decidualization of URSA, we compared the level of IGFBP1 and PRL by qRT-PCR, and determined the autophagy level by TEM, MDC assay and western blot in groups of ESC, DSC and 3-MA. The results showed that when compared with ESC group, the mRNA levels of IGFBP1 and PRL in DSC group were significantly increased (*p* < 0.01) (Figure 5A), and autophagosomes were increased under TEM (Figure 5B) and its fluorescence intensity by MDC assay (Figure 5C) was significantly elevated (*p* < 0.01). Besides, LC3II/LC3I ratio was increased (*p* < 0.05), and p62 was significantly decreased (*p* < 0.05) (Figure 5D), indicating that autophagy was activated after successful induction of decidualization *in vitro*. On the contrary, when compared with the DSC group, autophagy in the 3-MA group was effectively inhibited, as shown by the reduction of autophagosomes under TEM (Figure 5B) and the decreased fluorescence intensity by MDC assay (*p* < 0.01) (Figure 5C), and the decrease of LC3II/LC3I ratio and the significant increase of p62 (*p* < 0.05) (Figure 5D). Meanwhile, the mRNA levels of IGFBP1 and PRL were significantly decreased (*p* < 0.05) (Figure 5A), indicating that decidualization was directly affected after the inhibition of autophagy in DSCs.

### AMPK/mTOR/ULK1 Affected Decidualization *in vitro* by Mediating the Autophagy of Decidua Stromal Cells

**FIGURE 6:**
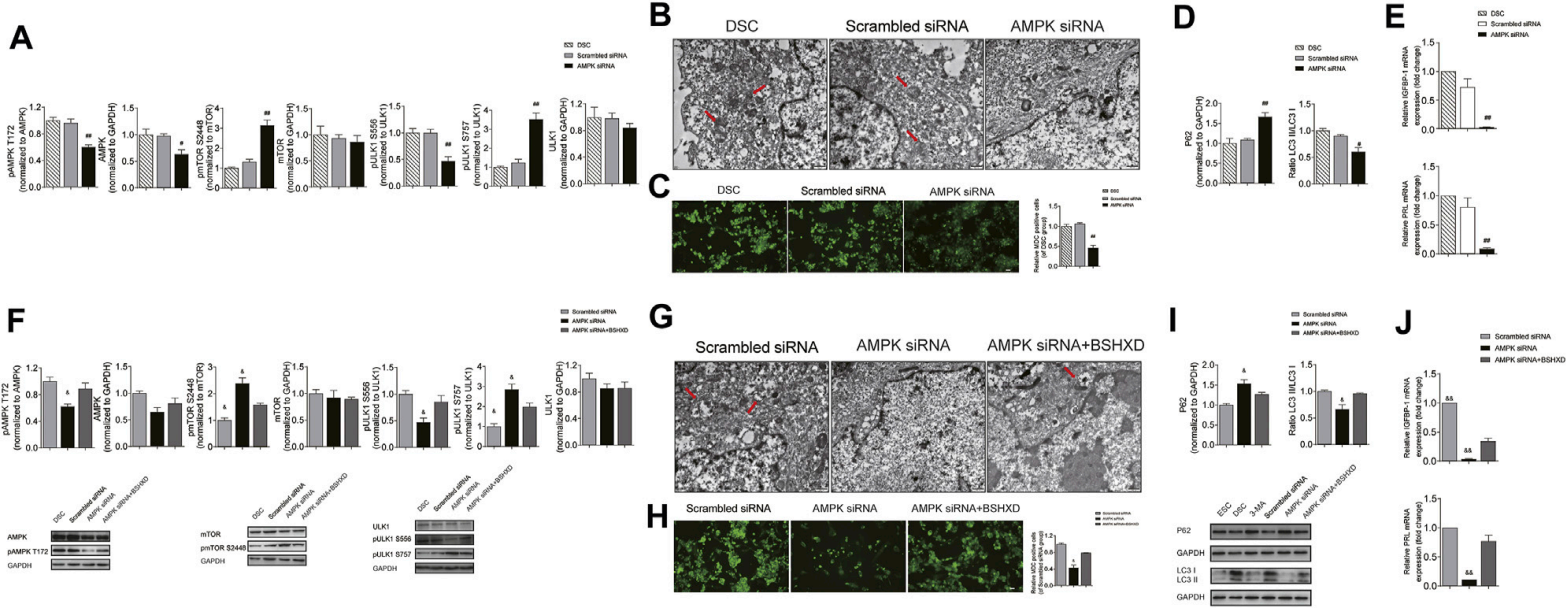
BSHXD improved decidualization by intervening autophagy via AMPK/mTOR/ULK1 in DSCs. **(A)** The expression levels of pAMPK, pmTOR and pULK1 in the DSC, Scrambled siRNA and AMPK siRNA group used by Western blot; **(B)** The autophagosomes in the DSC, Scrambled siRNA and AMPK siRNA group observed by TEM (scale bar: 500 nm); **(C)** The autophagy level in the DSC, Scrambled siRNA and AMPK siRNA group observed by MDC assay (scale bar: 50 μm); **(D)** The expression levels of LC3II/LC3I and p62 in the DSC, Scrambled siRNA and AMPK siRNA group used by Western blot; **(E)** mRNA of IGFBP1 and PRL in the DSC, Scrambled siRNA and AMPK siRNA group; **(F)** The expression levels of pAMPK, pmTOR and pULK1 in the Scrambled siRNA, AMPK siRNA and AMPK siRNA + BSHXD group used by Western blot; **(G)** The autophagosomes in the Scrambled siRNA, AMPK siRNA and AMPK siRNA + BSHXD group observed by TEM (scale bar: 500 nm); **(H)** The autophagy level in the Scrambled siRNA, AMPK siRNA and AMPK siRNA + BSHXD group observed by MDC assay (scale bar: 50 μm); **(I)** The expression levels of LC3II/LC3I and p62 in the Scrambled siRNA, AMPK siRNA and AMPK siRNA + BSHXD group used by Western blot; **(J)** mRNA of IGFBP1 and PRL in the Scrambled siRNA, AMPK siRNA and AMPK siRNA + BSHXD group. ^#^ *p* < 0.05 vs. Scrambled siRNA group; ^##^ *p* < 0.01 vs. Scrambled siRNA group. ^&^ *p* < 0.05 vs. AMPK siRNA + BSHXD group; ^&&^ *p* < 0.01 vs. AMPK siRNA + BSHXD group.

To further clarify whether AMPK regulates autophagy in decidualization by affecting the activation of mTOR and ULK1, we compared the phosphorylation of AMPK/mTOR/ULK1, the level of autophagy and decidualization markers in groups of DSC, AMPK siRNA and scrambled siRNA. Results showed that in AMPK siRNA group, the level of pAMPK T172/AMPK and pULK1 S556/ULK1 was decreased (*p* < 0.01), and the level of pmTOR S2448/pmTOR and pULK1 S757/ULK1 was increased when compared with the scrambled siRNA group (*p* < 0.01) (Figure 6A). Moreover, the number of autophagosome detected by TEM (Figure 6B) and the fluorescence intensity of autophagosome by MDC assay (Figure 6C) was significantly decreased in AMPK siRNA group when compared with the scrambled siRNA group (*p* < 0.01). Besides, the expression of p62 was increased and the ratio of LC3II/LC3I decreased in the AMPK siRNA group when compared with the scrambled siRNA group (*p* < 0.01 and *p* < 0.05, respectively) (Figure 6D). Furthermore, qRT-PCR showed that the mRNA levels of IGFBP1 and PRL in the AMPK siRNA group decreased significantly when compared with the scrambled siRNA group (*p* < 0.01) (Figure 6E). Besides, there was no significance in the phosphorylation of AMPK/mTOR/ULK1, the level of autophagy and decidualization markers between DSC group and scrambled siRNA group (Figures 6A–E). These results suggested that AMPK/mTOR/ULK1 mediated autophagy in DSCs and affected decidualization *in vitro*.

### Bushen Huoxue Decoction Improved Decidualization by Intervening Autophagy *via* AMPK/mTOR/ULK1

In order to clarify the regulation of BSHXD on AMPK/mTOR/ULK1, we compared the phosphorylation of AMPK/mTOR/ULK1, the level of autophagy and decidualization in groups of AMPK siRNA, AMPK siRNA + BSHXD and scrambled siRNA. The results showed that in AMPK siRNA + BSHXD group, the level of pAMPK/AMPK and pULK1 S556/ULK1 was increased, and the level of pmTORS2448/pmTOR and pULK1 S757/ULK1 was decreased when compared with AMPK siRNA group (*p* < 0.05) (Figure 6F). Moreover, the number of autophagosome detected by TEM (Figure 6G) and the fluorescence intensity of autophagosome by MDC assay (Figure 6H) were significantly increased in AMPK siRNA + BSHXD group when compared with the AMPK siRNA group (*p* < 0.05). Besides, the ratio of LC3II/LC3I increased and the expression of p62 was decreased in AMPK siRNA + BSHXD group when compared with the AMPK siRNA group (*p* < 0.05) (Figure 6I). Moreover, the mRNA levels of PRL and IGFBP1 were significantly increased in AMPK siRNA + BSHXD group when compared with AMPK siRNA group (*p* < 0.01) (Figure 6J). These results suggested that BSHXD can regulate the phosphorylation of AMPK/mTOR/ULK1 to affect the level of autophagy in DSCs and improve decidualization, which was conducive to the treatment of URSA. Moreover, there were no difference in pAMPK/AMPK and pULK1 S556/ULK1 between AMPK siRNA + BSHXD group and scrambled siRNA (*p* > 0.05), while pmTOR/mTOR and pULK1757/ULK1 in the AMPK siRNA + BSHXD group were significantly higher than those in the scrambled siRNA group (*p* < 0.05) (Figure 6F), suggesting that BSHXD can more effectively promote the direct phosphorylation of AMPK on ULK1 S556 to activate autophagy when compared with its promotion effect on AMPK phosphorylation to release the autophagy inhibition dominated by mTOR.

## Discussion

In this study, we observed impaired decidualization, decreased autophagy level and down-regulation of AMPK/mTOR/ULK1 pathway in the decidua tissue of URSA. Subsequently, we confirmed that the inhibition of autophagy can lead to impaired decidualization *in vitro*. And AMPK knockdown can inhibit autophagy by affecting the phosphorylation of mTOR and ULK1, thus affecting decidualization, while BSHXD can restore autophagy and improve decidualization by regulating AMPK/mTOR/ULK1. In summary, our study identified a novel mechanism of BSHXD in URSA treatment from the aspect of autophagy and provided a laboratory basis for its clinical promotion.

Decidualization provides growth factors and cytokines for embryo development and protects the blastocyst from immune rejection, which is a key prerequisite for embryo implantation and development (Zhang et al., 2019). Previous studies have shown that impaired decidualization is a crucial pathological link of URSA. For instance, Meng et al. found impaired decidualization in URSA featured with significantly decreased level of NDRG1 which was physiologically accumulated in the primary decidual region and contributed to the embryo implantation (Meng et al., 2019). In our studies, we also found impaired decidualization in URSA patients manifested by decreased mRNA levels of PRL and IGFBP1, which was consistent with the findings mentioned above. However, the molecular mechanism leading to impaired decidualization in URSA is still being explored.

The essence of decidualization is the transformation of fibroblast-like ESCs into secretory DSCs, leading to the decline of cellular ATP and intracellular remodeling, both of which are key signals for initiating autophagy (Buraschi et al., 2019; Ferro et al., 2020). In addition, autophagy contributes to the rapid clearance of mRNA and newborn proteins in response to the gene expression program in ESCs, contributing to complex gene expression changes during decidualization (Achache and Revel, 2006; He and Levine, 2010). Therefore, it can be concluded that moderate degree of autophagy ensures the progress of decidualization. In contrast, autophagy inhibition is probably related to impaired decidualization and adverse pregnancy outcome. In our studies, we took LC3II/LC3I and p62 as indicators to evaluate the level of autophagy. LC3 participates in the formation of autophagosomes. And stress sources can up-regulate the transformation of cytoplasmic LC3I to autophagose-specific LC3II, which suggests enhanced degree of autophagy (Oh et al., 2017). p62 can bind to the autophagosome membrane protein LC3 to transport the protein polymer to the autophagosome, and eventually be degraded by lysosomes (Katsuragi et al., 2015). Therefore, the decreased level of LC3II/LC3I and the increased p62 observed in the decidua tissue and DSCs of URSA represented the inhibition of autophagy (Jiang et al., 2018). This finding was consistent with Lu et al. who confirmed that the expression levels of autophagy related gene (ATG) 5 and MAP1LC3B were lower in DSCs of URSA, while the level of p62 was higher (Lu et al., 2020). Besides, we further confirmed the effect of autophagy on during induced decidualization *in vitro*. We displayed that the level of autophagy was increased in DSC group when compared with the ESC group, while the decidualization markers PRL and IGFBP1 in 3-MA group were significantly decreased after the successful inhibition of autophagy when compared with the DSC group. Therefore, it can be inferred that moderate level of autophagy are necessary for decidualization, which has also been confirmed by other researchers. Mestre et al. found that autophagy flux increased during decidualization (Mestre Citrinovitz et al., 2019), while decidualization was impaired in autophagy deficient cell models with ATG7 and ATG5 knockout (Mestre Citrinovitz et al., 2019). Su et al. observed that the expression of decidualization markers and the implantation sites were significantly reduced in mice treated with autophagy inhibitor (Su et al., 2020). Therefore, the decreased level of autophagy in URSA is one of the main reason that accounts for impaired decidualization. However, the regulation mechanism is very complex, and relevant researches are still scarce at present.

AMPK is a highly conserved serine/threonine isotrimer kinase in eukaryotic cells, and is widely involved in the metabolic process of various substances as well as in maintaining cell and systemic energy metabolism. It can activate autophagy and help with energy recycling by decomposing aging organelles and misfolded proteins so as to cope with energy stress and meet the high energy requirements of decidualization. mTOR and ULK1 are two important downstream factors of AMPK in the initiation of autophagy. mTOR is a vital regulator of cell growth, apoptosis, energy metabolism and autophagy as well as the growth of various tumors (Noda, 2017; Nnah et al., 2019). ULK1 is responsible for initiating autophagy, linking cellular nutrient status to downstream events in autophagy (Lin and Hurley, 2016). Literatures suggest that AMPK activation can phosphorylate tuberous sclerosis complex (TSC) 1/2 or directly phosphorylate raptor and prevent it from binding to mTORC1, thus preventing mTORC1 from phosphorylating ULK1 at Ser757 and removing its negative regulation of autophagy initiation (Wan et al., 2018). In addition, AMPK can also directly phosphorylate ULK1 at Ser556(Spengler et al., 2020) and induces an increase in autophagy (Egan et al., 2011). Immunohistochemical analysis in our study showed that the positive area of pAMPK T172 and pULK1 S556 in URSA group was less than that in control group, while pmTORS2448 and pULK1S757 was more than that of control group. Besides, immunofluorescence showed that pAMPK T172 and vimentin were colocalization, indicating that the activation level of AMPK in DSCs was reduced, which was consistent with the decreased level of autophagy in URSA. The above experiments jointly indicated that the decreased autophagy of DSCs in URSA may be mediated by AMPK/mTOR/ULK1 signaling pathway. To further confirm this hypothesis, we transfected AMPK siRNA in ESC during induced decidualization *in vitro*. The results showed that pAMPK/AMPK and pULK1 S556/ULK1 were significantly decreased in AMPK siRNA group when compared with the scrambled siRNA group, while pmTOR S2448/mTOR and pULK1 S757/ULK1 were significantly decreased in AMPK siRNA group. And, as a result, the levels of autophagy and decidualization were correspondingly decreased. Combined with the above finding on the relationship between autophagy and decidualization, we concluded that AMPK/mTOR/ULK1 can affect decidualization by regulating autophagy.

Next, we focused on the regulation mechanism of BSHXD on decidualization from the aspect of autophagy mediated by AMPK/mTOR/ULK1. During decidualization *in vitro*, we treated cells with BSHXD after transfection with AMPK siRNA and compared it with AMPK siRNA group and scrambled siRNA group. The results showed that BSHXD significantly increased the levels of pAMPK/AMPK and pULK1 S556/ULK1, degraded the level of pmTOR S2448 and pULK1 S757/ULK1, enhanced the level of autophagy, and improved decidualization impaired by AMPK knockdown. Therefore, we speculated that BSHXD could improve autophagy by regulating AMPK/mTOR/ULK1, thus contributing to decidualization *in vitro*. In addition, when we compared phosphorylation state of AMPK/mTOR/ULK1 between AMPK siRNA + BSHXD group and scrambled siRNA group, we found that ULK1 S556 was more sensitive to AMPK phosphorylation mediated by BSHXD than mTOR. That was to say, the regulation effect of BSHXD in AMPK/pULK1 S556 was superior to AMPK/pmTOR S2448/pULK1 S757. The possible explanation for this phenomenon is that the phosphorylation of mTOR is affected by multiple factors, and BSHXD is multi-targeted. And some upstream factors of mTOR may interfere with its phosphorylation by AMPK under the influence of BSHXD. However, this speculation needs further confirmation.

**FIGURE 7:**
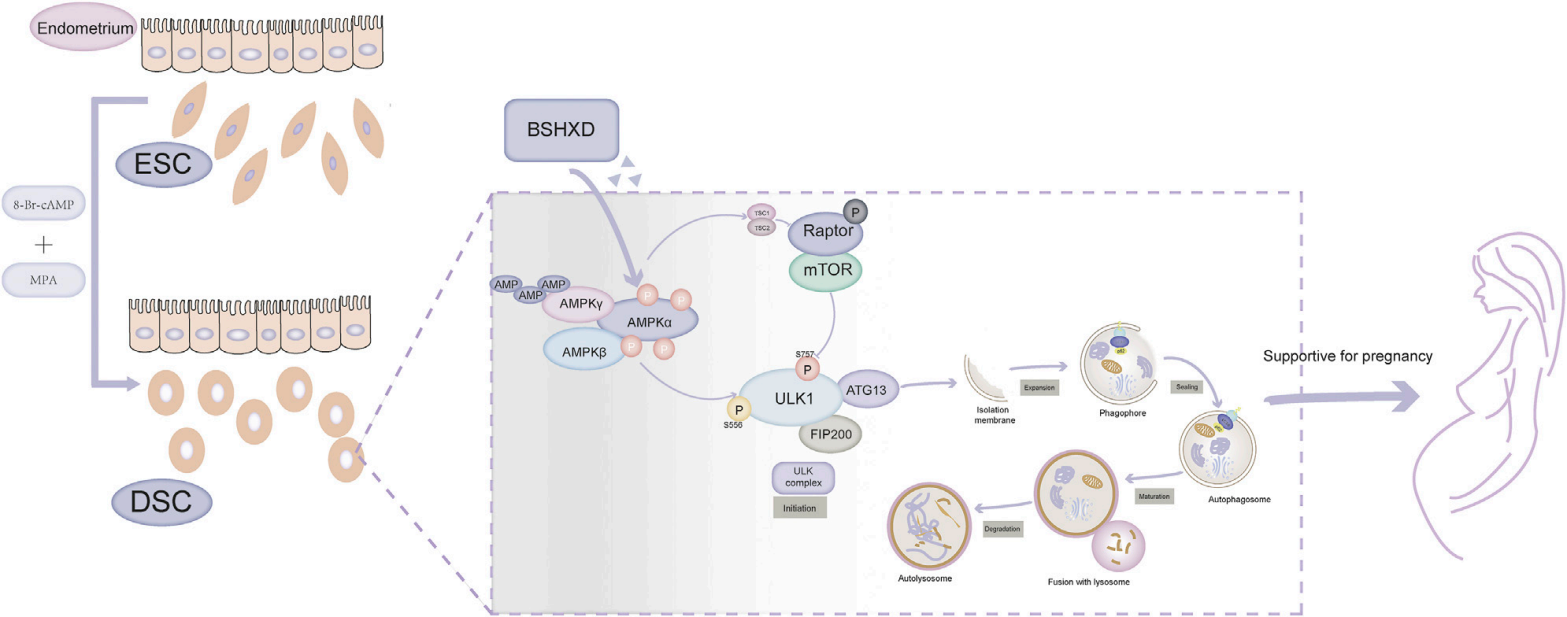
The mechanism of BSHXD on decidualization by intervening autophagy *via* AMPK/mTOR/ULK1.

Taken together, we concluded that BSHXD improved decidualization by activating autophagy via AMPK/mTOR/ULK1 signaling pathway, thus rationalizing its potential as a novel therapeutic regime for impaired decidualization and pregancy loss (Figure 7). In future experiments, molecular mechanisms that account for the decreased level of pAMPK in URSA need to be further demonstrated by bioinformatics techniques and experiments. Besides, animal experiments are necessary to confirm the influence of autophagy on embryo implantation rate and absorption rate mediated by AMPK/mTOR/ULK1, and further detect the active ingredients, the targets and the mechanism of BSHXD on this process *in vivo*.

## Data Availability

The original contributions presented in the study are included in the article/Supplementary Material, further inquiries can be directed to the corresponding author.

## Supplementary Material

Supplementary Figure S1 The information obtained by secondary mass spectrometry.

Supplementary Figure S2 CCK-8 assay was utilized to calculate the cell viability of each group.

Supplementary Figure S3 Selection of the AMPK siRNA with best knockdown efficacy. **p* < 0.05 vs. Scrambled siRNA group; ***p* < 0.01 vs. Scrambled siRNA group.
