# Supplementary figures and images for: Deciphering the Mechanism of Bushen Huoxue Decotion on Decidualization by Intervening Autophagy via AMPK/mTOR/ULK1: A Novel Discovery for URSA Treatment
Source: Front Pharmacol. 2022 Jan 24;13:794938. doi: 10.3389/fphar.2022.794938 (PMC8819596; doi:10.3389/fphar.2022.794938)

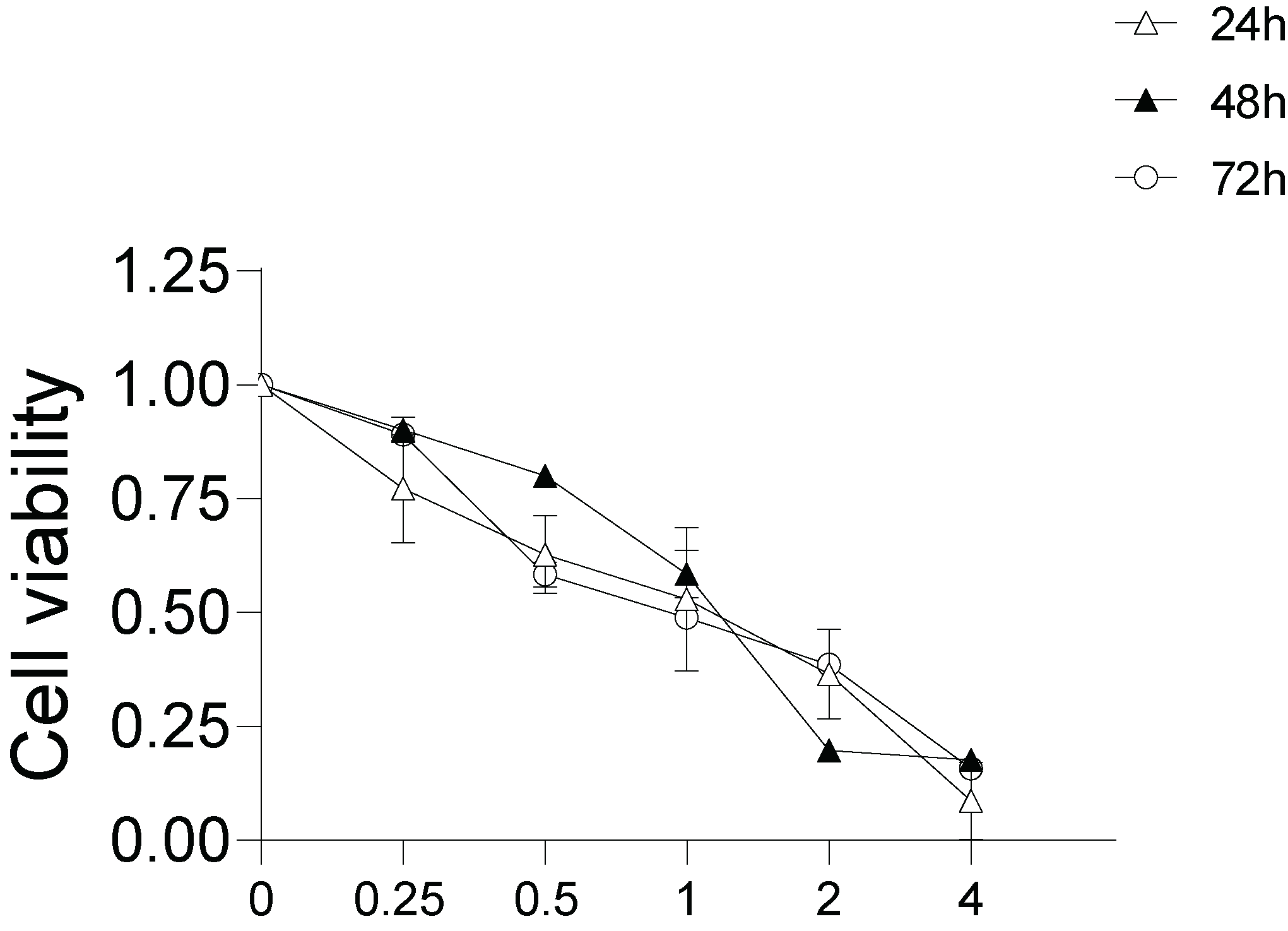

Supplement: Supplementary file 2 [file Image2.TIF]

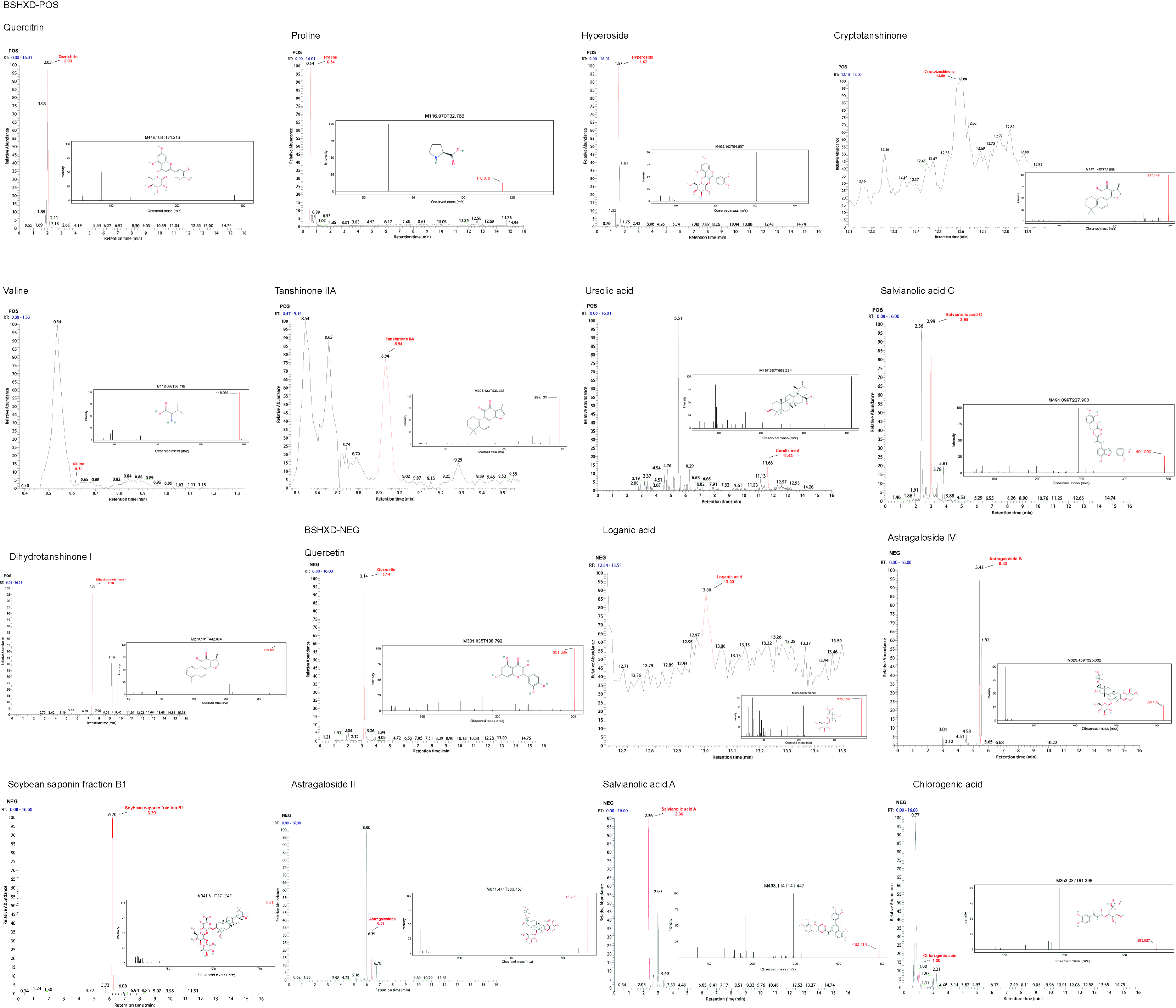

Supplement: Supplementary file 3 [file Image1.TIF]
