# Supplementary material for: Deciphering the Mechanism of Bushen Huoxue Decotion on Decidualization by Intervening Autophagy via AMPK/mTOR/ULK1: A Novel Discovery for URSA Treatment
Source: Front Pharmacol. 2022 Jan 24;13:794938. doi: 10.3389/fphar.2022.794938 (PMC8819596; doi:10.3389/fphar.2022.794938)
Supplement: Supplementary file 4 [file Table1.doc]

Supplement Table 1. Baseline characteristics of participating patients

|  | Control Group（n=14） | URSA Group（n=16） | P |
| --- | --- | --- | --- |
| Age (years) | 30.143±4.639 | 30.688±2.414 | 0.697 |
| The gestation period (days) | 56.00（46.75-60.50） | 55.00（50.50-59.00） | 0.918 |
| BMI | 21.410±1.475 | 21.718±2.178 | 0.658 |
| The menstrual cycle (days) | 28.500±2.175 | 27.938±1.389 | 0.400 |
